# Supplementary material for: Reconstruction of the ancestral metazoan genome reveals an increase in genomic novelty
Source: Nat Commun. 2018 Apr 30;9:1730. doi: 10.1038/s41467-018-04136-5 (PMC5928047; doi:10.1038/s41467-018-04136-5)
Supplement: Supplementary file 1 — Supplementary Information [file 41467_2018_4136_MOESM1_ESM.pdf]

Supplementary Note for

Reconstruction of the ancestral metazoan genome reveals an increase of genomic novelty

Paps & Holland

Table of contents

Supplementary Note 1. Types of lists of homology groups ... 3

Ancestral homology groups ... 3

Ancestral Core homology groups ... 3

Novel homology groups ... 4

Novel Core homology groups ... 5

Lost homology groups ... 6

Supplementary Note 2. False negatives and positives ... 8

False negatives ... 8

False positives ... 9

Supplementary Note 3. Percentages ... 11

Supplementary Figures ... 13

Supplementary Note 1. Types of lists of homology groups

Ancestral homology groups

Reconstruction of the complete complement of homology groups present in the last common ancestor (LCA) of a taxonomic group. These are inferred by mining for the homology groups present in at least one genome from the in-group lineage positioned as sister group to the rest of the clade and at least one genome of the other clade lineages (Supplementary Data 2). For example, the list of Ancestral groups of homology in metazoans would comprise the clusters present in at least one poriferan genome and one other metazoan representative, under a scenario where Porifera are the sister group to all other Metazoa.

This list is sensitive to the phylogeny used, as using a different first-lineage will produce different results. For example, the position of Porifera as sister group to the other metazoans will produce a different list of homology clusters in the metazoan LCA compared to placing Ctenophora in the same node. Some of those homology groups will be present in older nodes (e.g. Ancestral genes in the LCA of Metazoa may be found also in the LCA of Opisthokonta, such as TALE homeobox genes). Some clusters will be shared with younger LCA (i.e. Ancestral genes in the LCA of Metazoa may be found in later nodes if they have not been evolutionarily lost in intermediate nodes, e.g. the Wnt genes).

Ancestral Core homology groups

Subset of the ancestral complement present in the LCA of a clade (Ancestral, see above) that embraces homology groups never lost in any of its descendants, or only

absent in a single terminal branch/species; the latter is to accommodate clusters whose genes may be missing due genome assembly/annotation errors, producing technical false negatives. They are inferred by mining for the homology clusters present in all the members of the clade, or found in all members except for a single representative (Supplementary Data 2). For example, the list of Ancestral Core HG in metazoans would comprise homology groups found in all metazoans plus clusters present in all metazoans minus one species; in other words, homology groups present in at least 41 metazoan genomes out of the 42 sampled.

Ancestral Core lists are not sensitive to the phylogeny used, as the homology groups extracted are present in all members of the clade independently of the identity of its first-lineage. For example, the position of Porifera or Ctenophora as sister group to the rest of the metazoans will have no impact as the list contains clusters found in all the metazoan genomes (or all metazoans minus one terminal branch). Some of those homology groups may be present in older LCA (e.g. some of the clusters present in all Metazoa may be shared with all Opisthokonta). All the homology groups will be present in younger LCA (e.g. clusters present in all Opisthokonta must be also be present in all Metazoa).

Novel homology groups

Subset of the complement present in the LCA of a taxonomic group (Ancestral) that contains only those homology groups gained at the LCA node; these are therefore considered novelties. They are inferred by mining for the homology groups present in at least one genome from the in-group lineage positioned as sister group to the rest of

the clade plus at least one genome of the other clade lineages, but absent in all the outgroups (Supplementary Data 2). For example, the list of Novel homology groups in metazoans would include the clusters present in at least one poriferan genome and one other metazoan representative, but absent in all the non-metazoan eukaryotes (under a model where Porifera are the sister group to all other Metazoa). This list is sensitive to the phylogeny used, as using a different sister lineage will produce different results (see Ancestral above).

By definition, none of those homology groups will be present in complements of older nodes (e.g. novelties of the LCA of Metazoa will not be found in the LCA of Opisthokonta), ancestral or novel. They will not be part of the novel complement of younger nodes either (i.e. novelties of Metazoa are novelties only for Metazoa and not for Bilateria). However, they may be present in the ancestral complements of younger LCA (i.e. novelties in the LCA of Metazoa may be present in the ancestral complement of the LCA of Bilateria, e.g. Wnt genes).

Novel Core homology groups

Subset of the novel complement present in the LCA of a clade (Novel) that includes homology groups never lost in any of its descendants, or only lost in a single terminal branch/species (comparable logic to 'Ancestral Core' above). They are inferred by mining for the homology clusters present in all the members of the clade, or just missing in a single representative, but absent in the outgroup (Supplementary Data 2). For example, the list of Novel Core homology groups in metazoans would comprise clusters found in all metazoans, or present in all metazoans minus one

species, but absent in all non-metazoan eukaryotes; in other words, homology groups present in at least 41 metazoan genomes out of the 42 sampled and missing in all the outgroups.

Novel Core gene lists are not sensitive to the phylogeny used (as with 'Ancestral Core' above). As in the case of Novel homology clusters, none of those groups will be present in any clusters of older nodes (ancestral or novel) or the novel clusters in younger nodes, since novelties cannot be older than their node of origin.

Lost homology groups

Reconstruction of the complete of homology groups lost in the last common ancestor (LCA) of a taxonomic group. These are inferred by mining for the homology groups present in at least one genome from the in-group lineage positioned as sister group to the rest of the clade and at least one genome of the other clade lineages (Supplementary Data 2). For example, the list of Ancestral groups of homology in metazoans would comprise the clusters present in at least one poriferan genome and one other metazoan representative (under a model where Porifera are sister group to all other Metazoa).

This list is sensitive to the phylogeny used, as using a different first-lineage will produce different results. For example, the position of Porifera as sister group to other metazoans will produce a different list of homology clusters in the metazoan LCA compared to placing Ctenophora in the same node.

Some of those homology groups will be present in older nodes (e.g. Ancestral genes in the LCA of Metazoa may be found also in the LCA of Opisthokonta, such as TALE homeobox genes). Some clusters will be shared with younger LCA (i.e. Ancestral genes in the LCA of Metazoa may be found in later nodes if they have not been evolutionarily lost in intermediate nodes, e.g. the Wnt genes).

Supplementary Note 2. False negatives and positives in the lists of homology groups

False negatives

A false negative is defined as a homology group that should have been assigned to a given node, but it is missing in the list of clusters for that node.

False negatives could be caused by the homology assignment algorithm clustering genes in the wrong homology groups. For example, a false negative in the LCA of Metazoa could be caused by the clustering failing to assign all poriferan genes of a homology group in their proper metazoan-specific cluster, perhaps due to excessive sequence divergence. This would artefactually push the origin of the cluster one node younger (Eumetazoa): hence, a false negative in the LCA of Metazoa and false positive in the LCA of Eumetazoa (defined as Metazoa except sponges, under a 'Porifera basal' model).

False negatives can also be caused by sampling errors. Homology groups that were present in the LCA of a clade (ancestral or novel), but are absent in all the members of the in-group lineage positioned as sister group to the rest of the clade (e.g. in the case of Metazoa, clusters absent in all Porifera) are false negatives. They might be not found due to evolutionary loss, or because of sequencing/annotation errors. In the case of the lists of 'novel' groups, they will be mistakenly considered younger clusters (in the former example, groups gained in the LCA of Metazoa but absent in Porifera will be misclassified as of younger origin). Their assignment to a younger node will be a false positive for that node (see later).

The likelihood of false negatives is reduced by the fact that homology groups generally contain multiple genes per genome. For example, the homology group comprising Wnt genes ranges from 3 genes (in each poriferan genome) to 44 genes (in chicken). Thus, divergence or sampling errors would need to independently affect all the multiple genes of the same homology group across multiple genomes to produce a false negative, which is unlikely.

False positives

A false positive is defined as a homology group wrongly assigned to a given node, and that should not be present in the list of clusters for that node. In some cases, a false negative for one node will become a false positive for another.

False positives can be caused by the assignment algorithm assigning genes to incorrect homology groups. For example, a false positive in the LCA of Metazoa could be caused by failure to assign a choanoflagellate homologue to a choanoflagellate-metazoan homology cluster; this would artefactually push the origin of the cluster to one node younger (false positive in the LCA of Metazoa and false positive in the LCA of choanoflagellates plus metazoans).

False positives can also be caused by sampling errors. For example, homology groups will become false positives for a given node if they are artefactually absent in the outgroups of that node. If they are missing in all the outgroups, it will affect novel lists; ancestral lists would be affected if homology groups are missed in only some of

the outgroups. Clusters might be missing due to sequencing/annotation errors, poor taxon sampling (a gene is missing in not-sampled outgroups), or even more unlikely, multiple recurrent evolutionary losses. The wide sampling, and focus on well-annotated genomes, will reduce the likelihood of these problems.

Supplementary Note 3. Percentages

Percentage of novelty: obtained by dividing the number of Novel homology groups by the Ancestral clusters (Supplementary data 5). For a given node, indicates the proportion of all the ancestral complement formed by novel clusters. For example, in the LCA of Metazoa the ancestral complement is formed by a total of 6331 homology groups (Ancestral), of which 1189 are Novel. Therefore, 18% of ancestral complement is novel.

Percentage of core gene types that are novel: obtained by dividing the number of Novel Core homology groups by the Ancestral Core clusters (Supplementary data 5). For a given node, indicates the proportion of core clusters in the ancestral complement (never lost or only lost once) that are novel. For example, the LCA of Metazoa complement contains 922 homology groups that have never been lost (or only once, Ancestral Core), of which 25 are novel (Novel Core). Therefore, 2.7% of the genes that are (almost) never lost are novelties of that clade.

Percentage of ancestral that is core: obtained by dividing the number of Ancestral Core homology groups by the Ancestral clusters (Supplementary data 5). For a given node, indicates the proportion of clusters that have never been lost (or only lost once) in the ancestral complement. For example, in the LCA of Metazoa the ancestral complement is formed by a total of 6331 homology groups (Ancestral), of which 922 are (almost) never lost (Ancestral Core). Therefore, 14% of the ancestral complement is refractory to gene loss.

Percentage of novelty that is core: obtained by dividing the number of Novel Core homology groups by the number of Novel clusters (Supplementary data 5). For a given node, this indicates the proportion of novel homology groups that have never been lost (or only lost once). For example, in the LCA of Metazoa the novel complement is formed by a total of 1189 homology groups (Novel), of which 25 are (almost) never lost (Novel Core). Therefore, 2.1% of the novel complement is refractory to gene loss.

Supplementary Figures

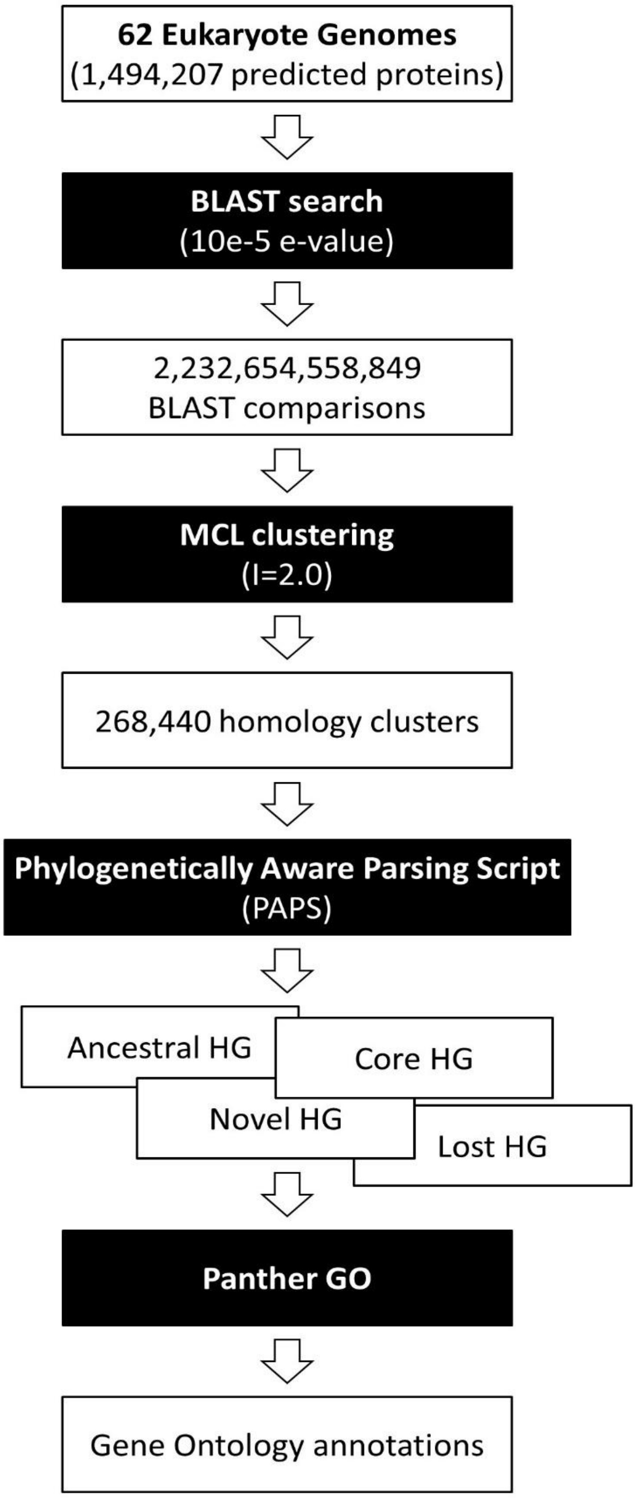

Supplementary Figure 1: Outline of the pipeline. White boxes indicate data inputs/outputs, black boxes programs used to analyse such data. See Methods for more details.

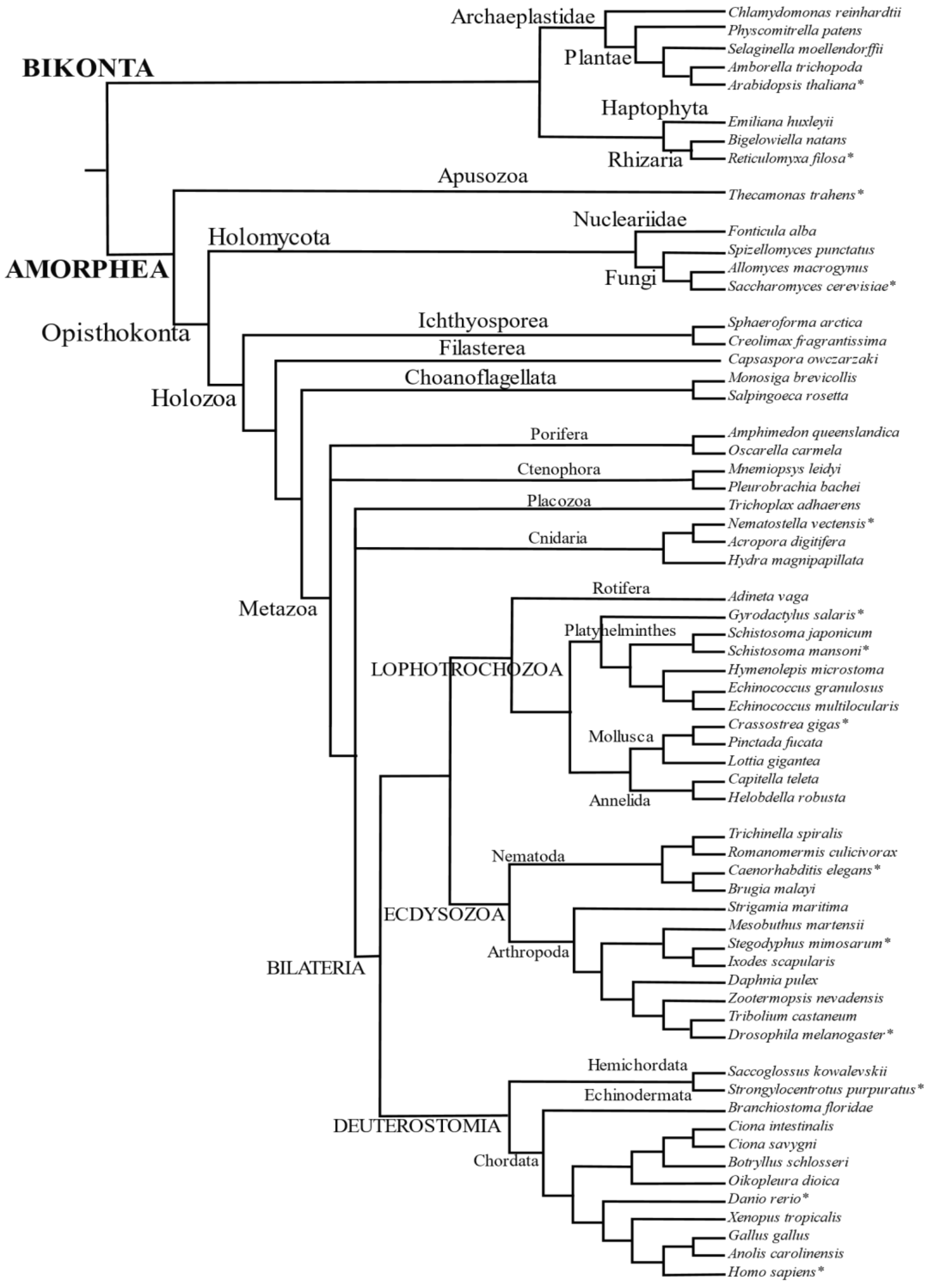

Supplementary Figure 2: Phylogeny of the taxa sampled at species level. Asterisks indicate genomes for which full gene annotations are available in our Dataet. See Methods for more details.

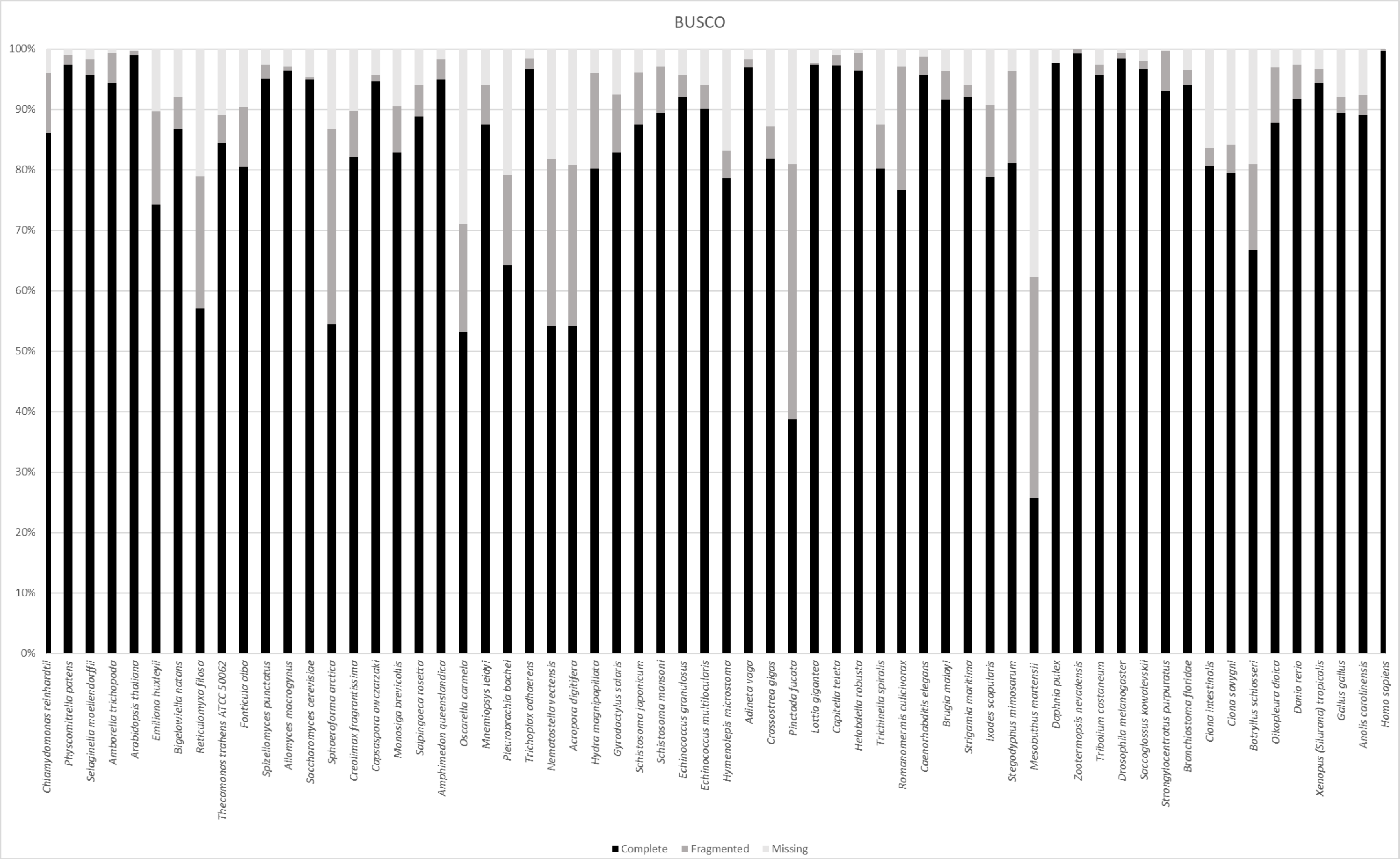

Supplementary Figure 3: Results of the BUSCO analyses for the genomes used in this study. See Methods for more details.
