# Supplementary material for: Reconstruction of the ancestral metazoan genome reveals an increase in genomic novelty
Source: Nat Commun. 2018 Apr 30;9:1730. doi: 10.1038/s41467-018-04136-5 (PMC5928047; doi:10.1038/s41467-018-04136-5)
Supplement: Supplementary file 3 — Description of Additional Supplementary Files [file 41467_2018_4136_MOESM3_ESM.pdf]

Description of Additional Supplementary Files

File Name: Supplementary Data 1
Description: List of the genomes sampled in this study, their BUSCO scores, and their respective sources.

File Name: Supplementary Data 2
Description: Different types of homology groups and their behaviour.

File Name: Supplementary Data 3
Description: Number of homology groups in each age category across different nodes of the Tree of Life of Eukaryota. This table analyses alternative phylogenetic scenarios regarding the nature of the first metazoan and early nodes.

File Name: Supplementary Data 4
Description: Gene ontology analyses of fruit fly genes belonging to Ancestral HG of the different Opisthokonta ancestors. This table analyses alternative phylogenetic scenarios regarding the nature of the first metazoan and early nodes.

File Name: Supplementary Data 5
Description: Percentages of different categories of genes across different nodes of the Tree of Eukaryota. This table analyses alternative phylogenetic scenarios regarding the nature of the first metazoan and early nodes

File Name: Supplementary Data 6
Description: Gene ontology analyses of the fruit fly genes belonging to Novel HG of the different Opisthokonta ancestors. This table analyses alternative phylogenetic scenarios regarding the nature of the first metazoan and early nodes. Each row contains different gene ontology categories, ordered following traditional ontology GO aspects (Protein Class, Molecular Function, etc.). Each LCA under consideration consists of two columns: the first contains the raw number of genes hitting that GO categories, the second the percentage these genes constitute respect the total of genes found under one ontology aspect. For the LCA of Metazoa, different evolutionary scenarios are explored based on alternative topologies.

File Name: Supplementary Data 7
Description: Representative genes of the Novel Core HG for the different annotated genomes in the pipeline. Each column contains the Novel Core HG for the LCA under investigation. The header of each HG is indicated in bold letters, followed by the gene descriptions from genomes with full gene annotations (indicated with asterisks in Supplementary Figure S2).

File Name: Supplementary Data 8

Description: Representative genes of the Lost HG for the different annotated genomes in the pipeline. Each column contains the Lost HG for the LCA under investigation. The header of each HG is indicated in bold letters, followed by the gene descriptions from genomes with full gene annotations (indicated with asterisks in Supplementary Figure S2).

File Name: Supplementary Data 9
Description: Results of the BLAST search to check the presence of false positives in the lists of Novel Core HG. BLAST searches were performed by using as query the fruit fly representatives for each of the Novel Core HG of all the clades of interest (Figure 1) against the complete NCBI GenBank database. Different folders correspond to different clades, each file contains the tabular output of the BLAST search of one HG.

File Name: Supplementary Data 10
Description: List of all the genes ID in the different HG categories for the different last common ancestors (see Figure 1). For each HG type (e.g. Metazoa Novel Core HG), two files can be found: 'annotated genes' provide a list of the genes coming from genomes with full gene annotation, while 'all genes IDs' gives a list of all the genes ID included in the HG (annotated and not annotated).
